# Electrochemical Activation of Fe-LiF Conversion Cathodes in Thin-Film Solid-State Batteries

**DOI:** 10.1021/acsnano.3c10146

**Published:** 2024-01-29

**Authors:** Joel Casella, Jȩdrzej Morzy, Evgeniia Gilshtein, Maksym Yarema, Moritz H. Futscher, Yaroslav E. Romanyuk

**Affiliations:** † Laboratory for Thin Films and Photovoltaics, Empa – Swiss Federal Laboratories for Materials Science and Technology, 8600 Dübendorf, Switzerland; ‡ Chemistry and Materials Design, Institute for Electronics,, Department of Information Technology and Electrical Engineering, ETH Zürich, 8092 Zürich, Switzerland

**Keywords:** Li-ion battery, solid-state battery, thin film, iron fluoride, conversion cathode, LiPON, battery

## Abstract

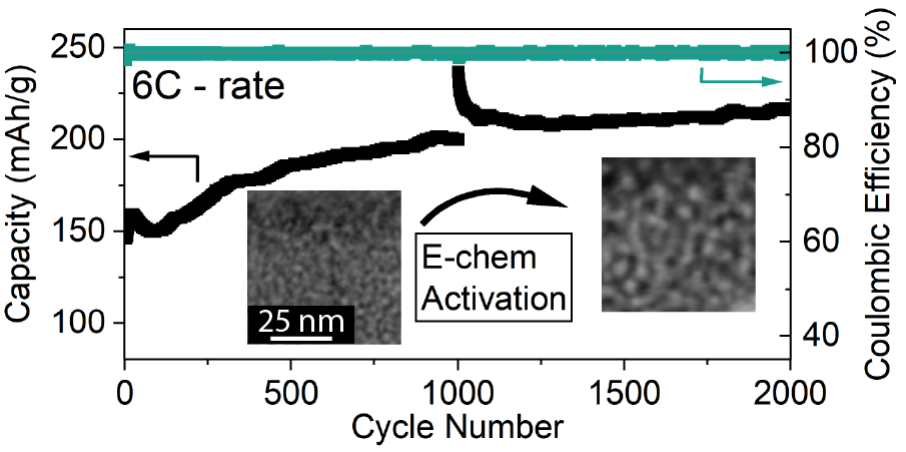

Transition metal fluoride (TMF) conversion-type cathodes promise up to 4 times higher gravimetric energy densities compared to those of common intercalation-type cathodes. However, TMF cathodes demonstrate sluggish kinetics, poor efficiencies, and incompatibility with many liquid electrolytes. In this work, coevaporated heterostructured iron and lithium fluoride (Fe-LiF) cathodes are investigated in thin-film solid-state batteries with a LiPON electrolyte and a lithium metal anode. The cells were cycled 2000 times at a cycling rate of 6C. They show a gradual improvement in voltaic efficiency (37–53%) and specific capacity (146–216 mAh/g) during cycling. After 2000 cycles, the cathode capacity reaches 480 mAh/g at a cycling rate of C/3.6, close to its theoretical capacity of 498 mAh/g, at room temperature conditions. This capacity gain is correlated with an observed electrochemically activated nanorestructuring of the cathode, characterized by cycling-induced coarsening (from 2.8 to 4.2 nm) of the metallic iron phase and its accumulation near the current collector interface, as well as lithium fluoride phase accumulation near the LiPON interface.

## Introduction

Intercalation cathodes are reaching their physicochemical power and energy density limits and hence, other cathode materials are being explored for lithium-ion batteries (LIBs).^1,2^ State-of-the-art intercalation-type positive electrodes (cathodes) mostly contain transition metal oxides (LiMeO_2_, LiMePO_4_, where Me = Fe, Co, Ni, Mn, etc.), and are widely used thanks to their cyclability and good power density.^3,4^ Transition-metal fluoride (TMF) cathodes which operate via a conversion reaction promise up to 4 times more gravimetric Li^+^ storage capacity than intercalation cathodes.^5^ FeF_3_ and FeF_2_ are by far the most studied TMF cathodes with theoretical energy densities when in charged states (delithiated) of 713 and 587 mAh/g and 2600 and 2363 mAh/cm^3^, respectively (FeF_*x*_ + *x*Li^+^ + *x*e^–^ ↔ Fe + *x*LiF, with *x* = 2 or 3).^6−10^ The theoretical capacities of the discharged (lithiated) products (Fe + 3LiF and Fe + 2LiF) are slightly lower at 604 and 498 mAh/g and 2202 and 2003 mAh/cm^3^, respectively (as calculated based on their molar mass). Comparatively, LiNi_0.8_Mn_0.1_Co_0.1_O_2_ (NMC811), a common cathode material for commercial LIB production, has a theoretical discharge capacity of 275 mAh/g (discharged state, calculated by removing 1 stoichiometric equivalent of Li^+^ from LiNi_0.8_Mn_0.1_Co_0.1_O_2_) and a practical capacity of ∼200 mAh/g (cycled to 4.2 V vs Li/Li^+^).^11,12^

However, TMF cathodes exhibit sluggish kinetics and poor reversibility, leading to difficulties in accessing all of their Li^+^ capacity. In practice, significant quantities of conductive additives are included in the cathode matrix (20–60% by weight of cathode layer) to achieve sufficient cathode utilization.^13,14^ In doing so, it can be difficult to study and understand pure material characteristics. In addition, TMF cathodes are plagued by poor stability versus many liquid electrolytes.^15,16^ In liquid electrolytes, cathode dissolution and ion shuttling have a detrimental effect on the capacity and rate performance of TMF cathodes.^13^ Furthermore, due to the volume and phase changes of such cathodes, solid electrolyte interface (SEI) stability and particle disintegration/fusing can also have a detrimental effect on capacity.^13^

Zhao et al. reported a lithiated iron fluoride cathode (1 Fe + 1 LiF) grown using pulsed laser deposition and could observe 350 mAh/g (41.6 mA/g current density) in a liquid electrolyte half-cell.^17^ Furthermore, Zhao et al. also showed a mixed metal cathode (1 Cu + 1 Fe + 2 LiF) with 420 mAh/g (41.6 mA/g current density) capacity with 88% capacity retention after 200 cycles (95% coulombic efficiency).

In this work, a 1 Fe + 2 LiF thin-film cathode was grown by thermal coevaporation. Using thin-film deposition techniques, well-controlled and pure materials can be used to create model cell systems that do not require any additives. In this way, the electrochemical activity of the TMF cathodes can be isolated. This cathode was cycled against a Li-metal anode, using a LiPON solid electrolyte. LiPON had been selected due to its stability in a wide potential window.^18^ The Fe-LiF cathode was investigated electrochemically in order to better understand how it behaves in contact with LiPON. An electrochemically driven activation mechanism is observed at certain cycling rates connected to a change in microstructure that is studied through microscopy.

## Results and Discussion

**Figure 1:**
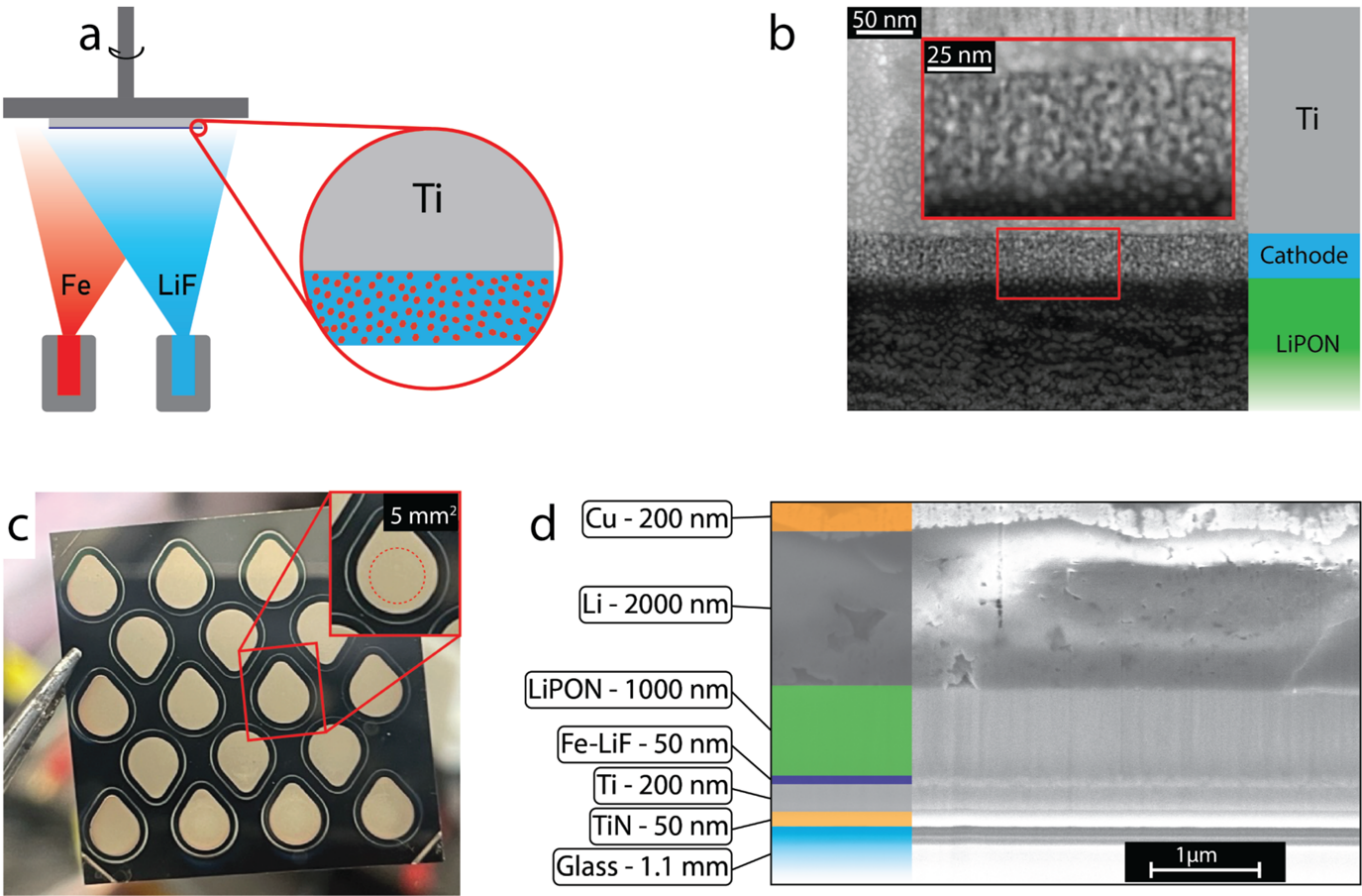
**Fe-LiF cathode and solid-state cell structure** (a) Schematic of the coevaporation step conducted for fabrication of Fe-LiF cathodes. (b) HAADF-STEM micrograph of the as-deposited cathode on a Ti (200 nm) and TiN (50 nm) coated glass substrate. (c) Photograph of the as-deposited array of thin-film cells. The inset shows an enlarged view of a single cell, and the dotted red line indicates the cathode active area (5 mm^2^). (d) FIB-SEM image of a cell cross section.

Coevaporation was used to create a well-mixed, heterogeneous 2-phase system of Fe and LiF as the cathode (see Figure 1a). Cathode thickness was varied depending on the requirements for different experiments, but the deposition conditions (deposition rates, substrate temperature, rotation speed, etc.) are kept constant. The high-angle annular dark-field scanning transmission electron microscopy (HAADF-STEM) micrograph (Figure 1b) shows darker and brighter regions, indicating Li- and F-rich domains and Fe-rich domains, respectively. The layer thickness is mostly constant throughout the imaged area. The Fe-rich nanodomains measure roughly 2–3 nm in diameter (see Figure S1). This nanodomain size is likely related to the deposition rate of the layers and could be tuned to achieve different cathode characteristics. Figure 1c is a top-view photograph of 18 fully assembled thin-film solid-state cells on a Ti (200 nm thick) and TiN (50 nm thick) coated glass substrate measuring 25 × 25 mm^2^. The inset image depicts a close-up of one cell and where the cathode was placed within the structure (embedded under subsequent layers; red dotted line). The cathode is circular, with an active area of 5 mm^2^ (2.73 mm cathode diameter). The cathode was then fully covered by a LiPON electrolyte (1000 nm thick, outermost edge of the cell structure). Finally, an anode (2000 nm thick Li and 200 nm thick Cu) was deposited in the inset from the electrolyte layer to avoid short circuiting to the Ti layer, but with an area larger than the cathode area to ensure full cathode activity (gold-colored layer in Figure 1c). 2000 nm thick metallic Li was used as a Li reservoir that also provides a stable electrochemical potential in order to isolate cathode electrochemistry. All layers are also shown with cross-sectional SEM micrographs in Figure 1d. XPS and XRD measurements were conducted to determine the chemical state and the crystallography. The results confirm the presence of Fe metal and LiF salt within the cathode matrix through their respective crystallographic peaks and binding energies (see Figures S2 and S3 and Supplementary Note 1). Cathode composition was controlled during deposition by calibrated independent quartz-crystal microbalances measuring the deposition rates of Fe and LiF independently.

**Figure 2:**
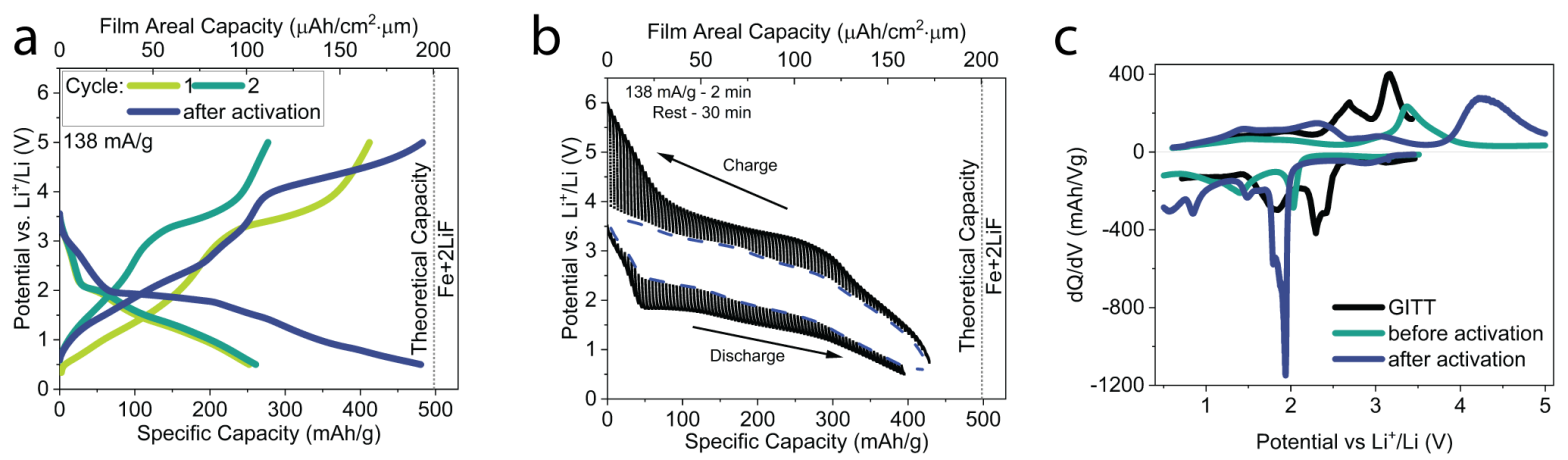
**Electrochemical characterization** (a) Charge–discharge curves of the first, second, and after electrochemical activation cycle of a Fe-LiF cell measured at 138 mA/g (roughly C/3.6 vs theoretical capacity). (b) GITT measurement results. The blue dashed line indicates the pseudoequilibrium voltage profile of the cell as measured from the data points after each rest phase. (c) d*Q*/d*V* curves as calculated by the pseudoequilibrium voltage profile obtained from the GITT measurement and the voltage profile of the second and after activation cycles in (a).

For this work, cut-off voltages for charging and discharging the cells were 5 and 0.5 V (vs Li^+^/Li), respectively. This range was chosen in order to maximize TMF cathode utilization while still maintaining LiPON electrolyte stability (Figure S4). Above 5 V, the side reactions of the electrolyte become significant, leading to considerable cell degradation over long-term cycling. For voltages below 0.5 V, there is no significant capacity contribution, thus falling outside the optimal operational range for the battery. Setting the cutoff voltage at 0.5 V also helps in circumventing issues related to Li alloying or plating on the cathode side. The typical voltage profiles of the cells are shown in Figure 2a. The low Coulombic efficiency (61.1%) during the first cycle is due to side reactions, which are likely to occur as the Li^+^ ions shuttle toward the anode, forming a solid–electrolyte interface at the LiPON–Li interface.^19^ In addition, overlithiation of the cathode (due to sputtering of the LiPON electrolyte) may also impact the low coulombic efficiency of the first cycle.^20^ The second cycle shows that the cathode discharge capacity is 261 mAh/g, roughly half of the theoretical capacity. This discrepancy is likely linked to poor kinetics within the cathode layer in its pristine state. It is well understood that Li^+^ conductivity in LiF is highly dependent on its crystal structure and favors an amorphous phase.^21^ As shown by Zhao et al., a mild post-deposition annealing process (200 °C for 2 h under high-vacuum) of similar cathodes improves the pristine lithiated cathode performance.^17^ In fact, after an activation protocol (5 cycles at C/8, 100 cycles at 6C, 5 cycles at C/8, and 100 cycles at 6C, explained in more detail later), the cell capacity is dramatically improved and reaches 480 mAh/g, very close to the expected theoretical capacity.

Figure 2b show the results of a galvanostatic intermittent titration technique (GITT) measurement performed with 2 min current pulses (same current density as in Figure 2a) and 30 min rest phases. In this technique, the kinetic and equilibrium phenomena can be deconvoluted and studied independently.^22^ Polarization is significantly impacted by the state-of-charge of the cell. This indicates that the charged and discharged states of the cathode exhibit dramatically different kinetic properties. This is also confirmed through electrochemical impedance spectroscopy (see Figure S5) where the cathode conductivity is calculated (through equivalent circuit fitting) to be 2.4 ± 0.1 × 10^–8^ and 1.3 ± 0.2 × 10^–10^ S/cm for the discharged and charged states respectively (errors represent one standard deviation of confidence). Hence, not only the reaction activation energy can affect cell overpotentials, but also the 2 orders of magnitude change in cathode conductivity between discharged and charged states. As shown by Figure2b, kinetic limitations of such thin TMF cathodes cannot be neglected. At a charging rate of 138 mA/g, overpotentials as high as 2 V are observed when the cathode is fully charged. The thin-film system allows us to precisely probe such kinetic limitations of pure cathode materials without having to consider the contribution of additives in composite cathode systems.

In the GITT experiment, the cathode demonstrates almost double the capacity as in the galvanostatic charging experiment for the pristine cathode state (Figure 2a) but similar values to the cathode after activation. Furthermore, a pseudoequilibrium voltage profile can be calculated, by interpolating the data points at the end of each rest phase. Subsequently, d*Q*/d*V* curves are calculated for both the galvanostatic (Figure 2a) and the pseudoequilibrium (Figure 2b) voltage profiles and are plotted in Figure 2c. The pseudoequilibrium d*Q*/d*V* (black line) indicates a multistep charging mechanism (Fe + *x*LiF → FeF_*x*_ + *x*Li^+^ + *x*e^–^, with *x* = 2 or 3), represented by the peaks at 2.69 and 3.20 V. The theoretical potentials for the conversion for FeF_2_ and FeF_3_ are 2.59 and 3.00 V respectively, closely matching the experimental results but also indicating the rest step in the GITT measurement did not reach equilibrium. In comparison, from galvanostatic cycling, d*Q*/d*V* curves were calculated for the second cycle, the peaks are shifted to higher voltages for charging due to kinetic considerations, and the cathode is showing minimal activity at the FeF_2_ peak, which could indicate preferential conversion directly to the FeF_3_ phase. This is likely due to local iron deficiencies at the reaction front due to its relatively poor mobility, as indicated by Li et al.^23^

During discharge, a peak shift toward lower voltage is observed for the pre-activation cathode as compared to the pseudoequilibrium curve. From the GITT measurements, the same features are observed. A minor peak is observed at 3.11 V attributed to intercalation of Li^+^ into the FeF_*x*_ phase before the conversion reaction occurs.^23^ The discharge peaks at 2.29 and 1.85 V are linked to the conversion reaction of the TMF cathode.^9^ Significant capacity is still observed at low voltage, but the voltage range was limited to 0.5 V in order to avoid the risk of Li-metal plating on the cathode at high charging/discharging rates in subsequent experiments.

After cathode activation, during charging, the principal capacity is occurring in a much higher potential region (4–5 V; Figure 2a). More capacity is also observed in the discharge curve, shifted to lower potentials by <0.1 V as compared to the preactivated cathode curve. This implies significantly better cathode utilization (the amount of cathode material is constant). These features are considered to be related directly to the conversion mechanism due to their very high respective capacities. Some features are also evident in the low voltage region (<1.5 V) for all d*Q*/d*V* curves, but the Li^+^ storage mechanism in this range is yet unclear and requires further studies.

**Figure 3:**
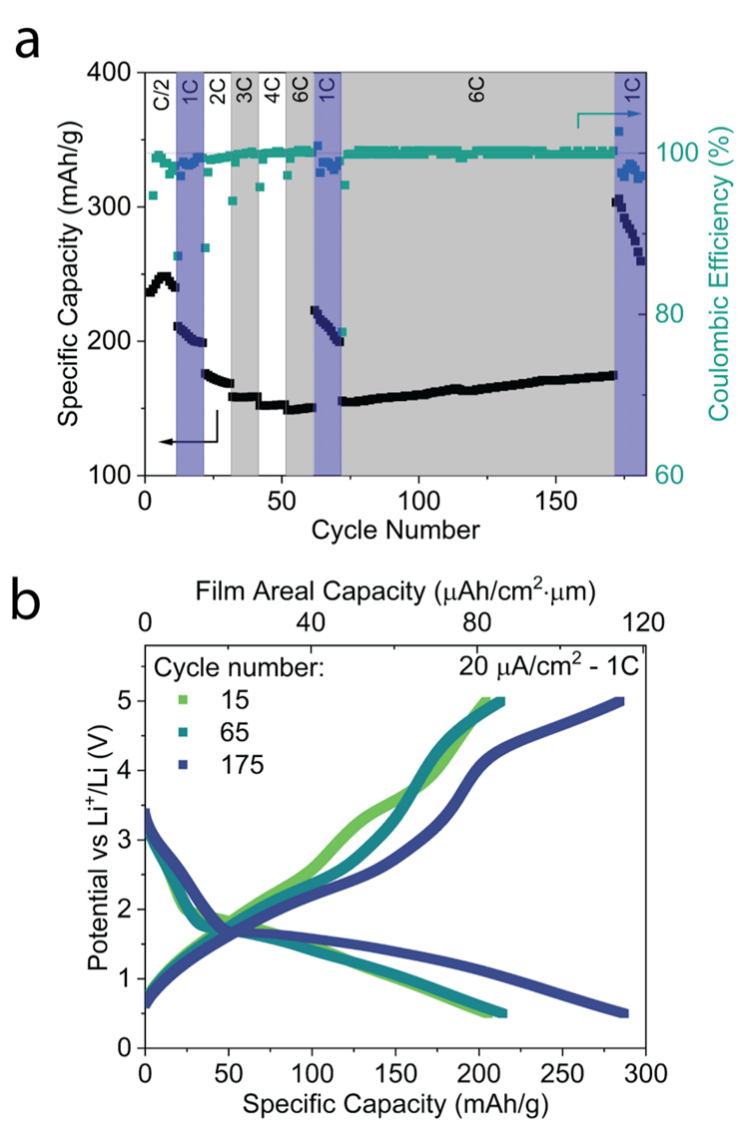
**High-rate cycling** (a) Specific discharge capacity of the Fe-LiF conversion cathode (90 nm thickness) at current densities ranging from 10 to 100 μA/cm^2^ (C/2 to 6C). Shaded blue regions compare 20 cycles of μA/cm^2^ (1C) at different stages of the experiment. (b) Charge–discharge curves of the 15th, 65th, and 175th cycles, all at a current density of 20 μA/cm^2^ (1C). C-rates are based on the theoretical capacity of FeF_2_.

The Fe-LiF cells were cycled at different C-rates in order to determine their rate capabilities (Figure 3a). As the C-rate is increased, discharge capacity decreases due to higher overpotentials which lead to less material conversion. At 6C (100 μA/cm^2^), cathode capacity was observed to increase from 154 to 174 mAh/g, depending on the cathode cycle number. Interestingly, this is a 13% capacity increase during the 100 cycles conducted at 6C. This experiment was conducted multiple times on different devices from the same substrate and from different batches, and a similar trend is observed in each case as shown in Figure S6. It is inferred that this effect is due to a rate-dependent nanorestructuring of the cathode during each charge and discharge. In theory, the cathode cycles between a 2-phase heterogeneous system (Fe + *x*LiF, discharged state) and a different, single phase system (FeF_*x*_, charged state), which implies a relationship between charging rate (a proxy for reaction rate) and the nanostructure of the product formed (both during charging and discharging). The restructured cathode can be beneficial to its performance by electrochemically activating more cathode regions, shortening diffusion pathways, and increasing the surface area for reactions. This is further validated by the discrete capacity slopes observed for each different charging rate. In the final 10 slower cycles (1C), a significant decrease in capacity is observed that may also be linked to a favorable nanostructure being replaced by a less favorable one when cycling at 1C (20 μA/cm^2^). In this way, cathode discharge capacity could be tuned as a function of the cycling rate and may be able to be regenerated if the cathode is cycled with an appropriate protocol, as previously shown by Le Cras et al. in a different cell system (Li_1.2_TiO_0.5_S_2.1_|LiPON|aSi).^24^ As the system relies on solid–solid (electro)chemical reactions, kinetic implications (for example, nucleation and growth of other phases) are key. Hence, it is thought that cycling at different rates will form different cathode nanostructures. This effect can be observed in the regions shaded in blue in Figure 3a. Over 10 cycles at 1C, the capacity is reduced each time at a different slope. The change in slope is likely due to the structure at the beginning of the 1C cycle set, determined by the C-rate of the preceding cycles. In this way, it can be concluded that the 1C cycles may reach a common “equilibrium” capacity after more cycles. Furthermore, it is evident that the restructuring takes place over more than 10 cycles (the slope in 1C regions does not reach a plateau, and longer 6C cycling results in higher capacity in the following 1C cycles). Figure 3b shows voltage profiles of the 15th, 65th, and 175th cycles corresponding to Figure 3a. A change in electrochemical activity is observed, leading to the formation of a new plateau at the high voltage region (4–5 V), indicating a reduction in overpotentials after 6C cycling allowing for more Li^+^ removal from the cathode, similar to the observation made in Figure 2c. Voltage plateaus in the region of 2.5 to 3 V are also extended during charging. During discharge, the largest improvement in capacity is indicated by the conversion reaction plateau (2 to 0.5 V) with some minor improvement to the higher voltage region (3.5 to 2 V), generally associated with the intercalation of Li^+^ into the FeF_*x*_ phase.^23^

**Figure 4:**
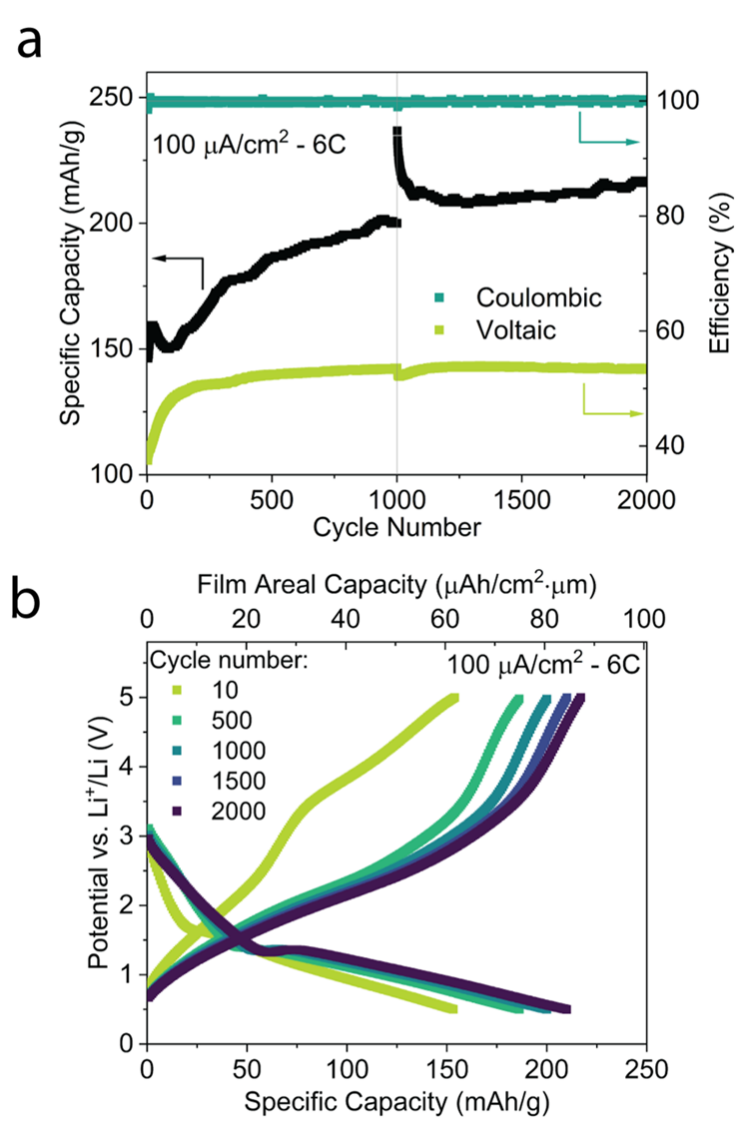
**Long-term cycling** (a) Specific discharge capacity (black) and respective coulombic and voltaic efficiency (teal and yellow) of Fe-LiF cell over 2000 cycles at a current density of 100 μA/cm^2^ (6C vs theoretical capacity of FeF_2_). A CV voltage sweep at a rate of 0.5 mV/s from 0.5 to 6 V was conducted before cycling and also between the 1000th and 1001st cycles. (b) Charge–discharge voltage curves of the 10th, 500th, 1000th, 1500th, and 2000th galvanostatic cycle, all at a current density of 100 μA/cm^2^.

Figure 4a shows cathode specific capacity, coulombic efficiency, and voltaic efficiency over 2000 cycles of the TMF cathode. A cyclic voltammetry (CV) voltage sweep (0.5 mV/s, 0.5 to 6 to 0.5 V) was conducted before cycling and then again after 1000 cycles (see Figure S7). During the 2000 cycle experiment, the cathode capacity increased from 146 to 216 mAh/g, a 48% increase. Furthermore, voltaic efficiency increased from 37 to 53% within the first 400 cycles. After the second CV voltage sweep, the capacity was initially significantly improved but quickly dropped back to follow the initial trend. This long-term increase in specific capacity during cycling further reinforces the hypothesis that the nanostructure affects the discharge capacity, as observed in Figure 3a and leads to a material “activation” enabling higher utilization of the cathode over time (30 to 43% utilization versus theoretical capacity at 6C). Another indication of this is that after the voltage sweep (with variable effective C-rate), capacity is greatly increased for a few cycles, likely due to full material conversion, but then settles back into the same positive trend of discharge capacity that is observed before the voltage sweep. This observation was reproduced by similar cells with the same Fe-LiF cathodes, this is shown in Figure S8. This positive trend in capacity indicates that there is an inherent advantage to converting the cathode material at high rates that is not observed if cathodes are cycled slower. Figure 4b shows voltage profiles related to different cycles from Figure 4a. Cycles 10, 500, 1000, 1500, and 2000 are shown. It is evident that the largest change in discharge capacity (and voltage profiles) is observed within the first 500 cycles, closely linked to the trend observed for the voltaic efficiency, as shown in Figure 4a. The voltage plateaus during charging indicate that significantly more Li^+^ is released from the cathode in the 1 to 3 V range in cycle 500 and beyond. During discharging, both the intercalation and conversion plateaus are extended in capacity after 500 cycles. In addition, a voltage “bounce back” is observed at 1.5 V for the later cycles, as discussed for iron fluoride conversion reaction mechanisms in the literature.^23^ This effect is attributed to the nucleation of Fe and LiF reaction products, which promote electronic and ionic diffusion to the reaction front, leading to improved kinetics and hence lower overpotentials.

**Figure 5:**
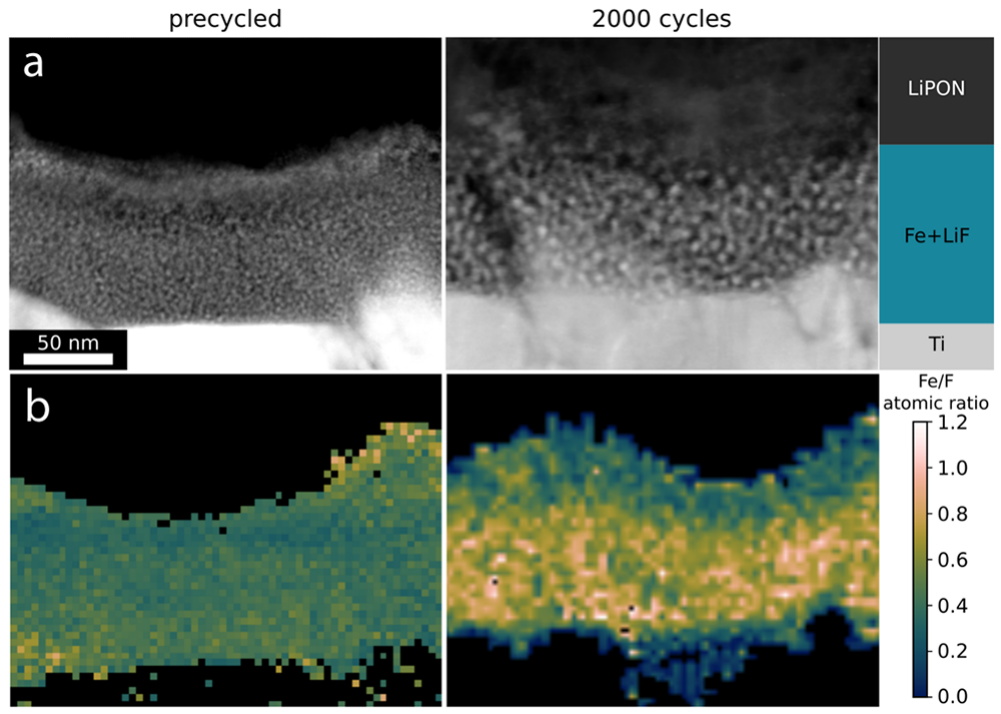
**Post-cycling characterization** (a) HAADF-STEM micrographs and (b) EDX atomic ratio Fe/F maps of Fe-LiF cathode after 10 cycles (precycled, left column) and after 2000 cycles (right column). Cathodes are imaged and measured in their discharged (lithiated) state. Cathodes were both discharged at 100 μA/cm^2^ (6C vs theoretical capacity of Fe-2LiF). The scale bar is shared between all panels.

Figure 5 shows HAADF-STEM micrographs and the corresponding measured Fe/F ratio as taken from EDX spectrum images in the precycled state (CV voltage sweep 0.5 mV/s from 0.5 to 6 V and then 10 cycles at 6C), and after 2000 cycles (6C). Similarly to Figure 1(b), the precycled cathode shows a well-mixed, two-phase system of Fe-rich nanodomains (2.8 nm in diameter, see Figure S1) and a F-rich matrix, which likely also contains the Li-ions in the form of LiF. This is further evident in the Fe/F ratio map, which indicates a well-mixed system for the two observed phases. After 2000 cycles at 6C, we observe a change in cathode nanostructure. First, a coarsening of the Fe-rich clusters is evident within the cathode structure, showing an increase in their average diameter from 2.8 to 4.2 nm (see Figure S1). Second, the Fe/F ratio map indicates an accumulation of Fe on the current collector-cathode interface and a similar accumulation of LiF on the electrolyte-cathode interface. No comment on the absolute values of the Fe/F stoichiometric ratios can be made due to inherent limitations of EDX (beam damage, limited field of view, etc.). In relation to Figure 4, it is clear that this electrochemically driven restructuring provides advantages to cycling performance. Further testing and characterization is necessary in order to determine the reason for this advantage. Due to the solid–solid nature of the chemical reactions taking place, a more evenly mixed cathode is, in theory, a preferred candidate allowing for shorter physical pathways for reactants to meet, but these data suggest otherwise. Studies on composite cathodes have shown that optimizing the cathode for roughly equal electronic and ionic conductivities leads to the highest cathode utilization.^25^ Following this theory, it is possible to suggest that the restructuring observed leads to favorable ionic and electronic conductive network. Notably, a gradient of Fe-LiF ratio is formed within the cathode during cycling (see Figure 5), with the highest electronic conductivity being near the current collector (due to aggregation of Fe near the current collector) and vice versa for the ionic conductivity (due to aggregation of LiF near the electrolyte). In the studied system, the LiF provides the role of the ionic conductor and Fe, the role of the electronic conductor in the discharged state. This may suggest a reason why the observed cycled cathode performs better than the pristine cathode.

Many works describe substantial complexities and issues associated with the use of liquid electrolytes and TMF cathodes.^13^ In addition, due to dramatic phase changes during TMF cathode cycling, composite cathode systems often struggle with material stability over many cycles, leading to SEI instability and capacity loss. After 2000 cycles at 6C, there is no observed capacity loss or evidence of cell degradation. Figure S9 shows EIS spectra taken every 50 cycles over the course of 1000 cycles. There is no observed degradation of the LiPON contribution. There is a decrease in the magnitude of impedance in the low frequency range, further solidifying the activation phenomenon described. Due to the thin nature of the system, any interface formation and/or degradation would significantly impact the cell’s performance. In contrast, an improvement of cell performance metrics is observed as the cells are cycled further. An activation of the cathode material is observed after such a cycling protocol that allows for very high cathode utilization (96%) with observed cathode gravimetric capacities of 480 mAh/g at a thickness of 90 nm.

## Conclusions

A thin-film solid-state battery was created with the scope of testing the electrochemical performance of Fe-LiF conversion cathodes with LiPON solid electrolytes. Using the thin-film solid-state system, 2000 cycles at 6C of cells with Fe-LiF cathodes have been achieved without noticeable degradation of the cell structure or performance. TMF cathodes are notoriously poorly compatible with liquid electrolytes and result in long-term irreversible capacity fading. Here, we show a Fe-LiF cathode with 480 mAh/g of capacity at a C/3.6 rate, 96% of theoretically achievable capacity after electrochemical activation. At 6C cycling rates, 216 mAh/g of discharge capacity remained after 2000 cycles. An important finding is that for such a coevaporated lithiated cathode system, an activation step is advantageous for higher cathode utilization, capacity, and improved energy efficiency. A distinct change in the nanostructure is observed which is likely linked to the cathode activation mechanism and improved cell performance metrics. Conversion cathodes still have a lot of untapped potential, and the thin-film system seems to be able to unlock some of it. As the rest of battery research is moving toward solid-state electrolytes, understanding how TMF cathodes interact in all solid-state cells will allow to better assess their usability in future generations of batteries.

## Experimental Section

### Fabrication

Uncoated Corning boro-aluminosilicate glass substrates (CB-0111, Delta Technologies, Ltd., 25 mm × 25 mm × 1.1 mm) were wiped clean with isopropyl alcohol and a lint-free tissue. Subsequently, TiN with a thickness of 50 nm was coated using a CT200 magnetron sputtering cluster (Alliance Concept) at 400 °C by DC magnetron sputtering of a 25 cm diameter target of Ti (gas flow of 120 sccm of Ar and 10 sccm of N_2_, working pressure of 3 mTorr) with a power of 3.1 W/cm^2^. Ti was then deposited with a thickness of 200 nm by using the same tool and target (21 sccm Ar gas flow, 2 W/cm^2^ power, and 3 mTorr pressure). The cathode was deposited to the desired thickness through a custom circular shadow mask (for 5 mm^2^ cells) using a Nextdep thermal evaporator (Angstrom Engineering Inc.) by coevaporating iron (042383.22, Thermo Fischer Scientific Inc.) and lithium fluoride (014463.18, Thermo Fischer Scientific Inc.) inside alumina crucibles (and a Ni crucible liner for LiF). The deposition rates of both materials were controlled during deposition using multiple quartz-crystal microbalances to achieve a stoichiometric ratio of 1:2 Fe:LiF. The solid electrolyte, LiPON was deposited to a thickness of 1000 nm through a custom electrolyte shadow mask using an Orion sputtering system (AJA International Inc.). The LiPON was deposited using RF cosputtering of Li_3_PO_4_ and Li_2_O 2” targets in a confocal arrangement at a gas flow of 50 sccm N_2_, a power of 4.93 and 5.92 W/cm^2^, respectively and a working pressure of 3 mTorr. The Li metal (211442500, Thermo Fischer Scientific Inc.) anode was deposited through a custom anode shadow mask using the same thermal evaporator as described above to a thickness of 2000 nm using an arc-coated stainless steel and alumina crucible. Finally, the anodic current collector (Cu, 010953.A1, Thermo Fischer Scientific Inc.) was deposited using the same anode shadow mask to a thickness of 200 nm through thermal evaporation.

### Characterization

Electrochemical characterization was performed in an Ar-filled glovebox at room temperature with no applied cell pressure using Squidstat Plus (EIS measurements) and Squidstat Prime (DC measurements) potentiostats (Admiral Instruments). The reported capacities correspond to electrode-level capacities calculated using real thickness, area, and calculated material densities. Applied currents range from 1 to 100 μA/cm^2^ in potential ranges from 0.5 to 5 V (vs Li^+^/Li). Cyclic voltammery was conducted between 0 and 6 V (1 mV/s scan rate) and 0.5 and 6 V (0.5 mV/s scan rate). Electrochemical impedance spectroscopy was done using a perturbation amplitude of 50 mV in a frequency range of 2 MHz to 0.5 Hz (12 steps per decade) after charging and discharging the cells at C/8.

X-ray photoelectron spectroscopy (XPS) measurements were performed by using a Quantum2000 photoelectron spectrometer from Physical Electronics with a monochromatic Al Kα source (1486.6 eV) and a base pressure below 8 × 10^–9^ mbar. High-resolution elemental spectra were recorded with an energy step size of 0.125 eV and a pass energy of 46.95 eV for Fe 2p and 29.35 eV for F 1s and Li 1s. Presputtering with 2 keV sputter energy was performed before spectra acquisition to get into the bulk of each sample. The estimated sputter depth for the Fe-LiF cathode sample is 20 nm, and 60 nm for Fe and LiF samples, due to the difference in the sample thicknesses. An ion neutralizer using Ar^+^ of ∼1 eV was used to minimize the fluctuations of the binding energy values due to possible sample charging.

X-ray diffraction experiments were conducted using a Bruker D8 Discover tool in grazing incidence mode using Cu Kα_1_ radiation at an incident angle of θ = 1° (for the Fe and LiF reference) and θ = 0.7° (for the cathode layer) and a measuring range of 2θ = 37–85°.

Samples for TEM were transferred to a Helios 660 (Thermo Fischer Scientific) FIB-SEM dual beam microscope with limited exposure to air (<1 min). The FIB-SEM was used to prepare TEM lamellae with a standard lift-out technique, but with an additional step of removing the Cu + Li layers at negative stage tilt to avoid issues with self-discharge and poor stability both mechanically and under the ion beam. The TEM lamellae were initially thinned using 30 kV Ga beam at a range of currents, followed by polishing at 8, 5, and 3 kV with lower ion beam currents until satisfactory electron beam transparency was achieved. The thinned lamellae were transferred to an Ar filled glovebox for storage and then to a TEM with total ambient air exposure <5 min. The TEM experiments were performed on a Talos F200X microscope (Thermo Fischer Scientific) operated at 200 kV accelerating voltage, equipped with a windowless Super-X EDS detector system. HAADF imaging and EDS spectrum imaging were performed in the scanning mode using Velox software. The EDS was performed by acquiring 60 frames and then assessing beam damage and choosing enough frames to ensure good signal-to-noise ratio while avoiding beam damage as much as possible. Pixel dwell time was kept at around 15 μs. Data processing to achieve the Fe/F ratio maps was done using open source Python package Hyperspy.^26^ First, the X-ray line intensity was calculated by numerically integrating the relevant X-ray lines. Then, the intensities were quantified to atomic % maps using the Cliff-Lorimer method using k-factors provided by the detector manufacturer. Fe and F atomic % maps were then divided by each other to arrive at the Fe/F atomic ratio maps. To measure the particle size distribution (PSD), HAADF images were cropped to the region of interest. Then, a Random Forest segmentation algorithm was trained to label the Fe particles and the LiF matrix using open source software Ilastik.^27^ The resulting binary masks were separated into particles using a watershed algorithm in ImageJ and last, their area was extracted and calculated into effective diameter assuming circular cross sections.^28^ The particle size distributions were fitted with a normal distribution.

## Supporting Information Available

Fe cluster sizes in nonactivated and activated cathode states; a note on XPS data analysis; XPS surveys of cathode and references; XRD diffractograms of the cathode and references; CV scans of cathode pre- and post-activation; EIS of cathode in charged and discharged state; Statistical validation for data presented in Figure 3; CV scans of cathode before and after 1000 cycles; Statistical validation of data presented in Figure 4; EIS measurements during long-term cycling experiment; Some references (PDF)
