# Supplementary material for: Electrochemical Activation of Fe-LiF Conversion Cathodes in Thin-Film Solid-State Batteries
Source: ACS Nano. 2024 Jan 29;18(5):4352–9. doi: 10.1021/acsnano.3c10146 (PMC10851659; doi:10.1021/acsnano.3c10146)
Supplement: Supplementary file 1 — nn3c10146_si_001.pdf [file nn3c10146_si_001.pdf]

Supplementary Information - Electrochemical activation of Fe-LiF conversion cathodes in thin-film solid-state batteries

Joel Casella,[*,†] Jędrzej Morzy,[†] Evgeniia Gilshtein,[†] Maksym Yarema,[‡] Moritz H. Futscher,[†] and Yaroslav E. Romanyuk[*,†]

†*Laboratory for Thin Films and Photovoltaics, Empa – Swiss Federal Laboratories for Materials Science and Technology, 8600 Dübendorf, Switzerland*

‡*Chemistry and Materials Design, Institute for Electronics, Department of Information Technology and Electrical Engineering, ETH Zürich, 8092 Zürich, Switzerland*

E-mail: joel.casella@empa.ch; yaroslav.romanyuk@empa.ch

Supporting Information Available

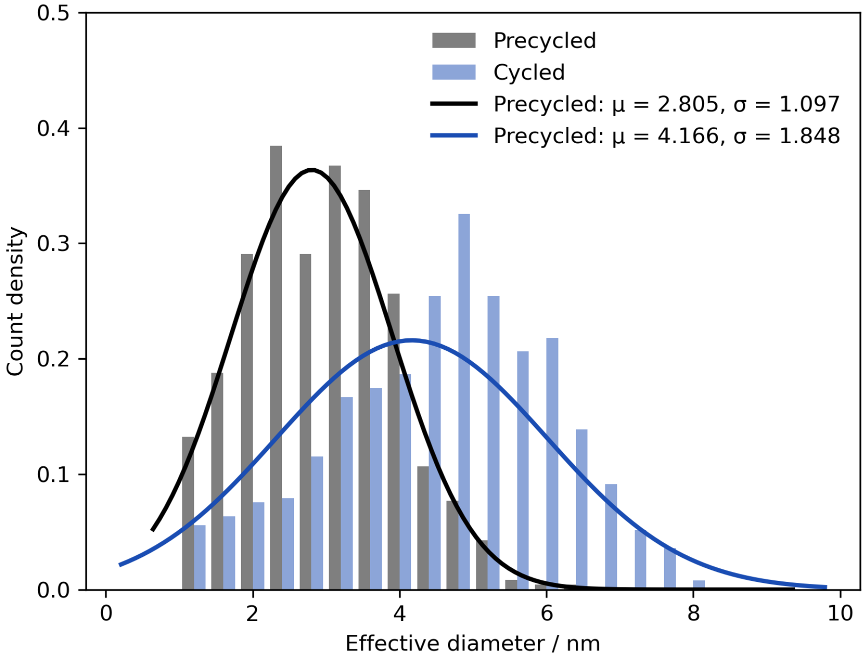

Figure S1: Fe cluster size distribution from precycled (CV and 10 cycles at 6C) and cycled (2000 cycles at 6C) cathodes.

Supplementary Note 1

Figure S2 show XPS spectra at different binding energies relating to characteristic peaks for Fe, Li and F. Reference spectra were measured using in-house references created using the same evaporation technique and materials and measured during the same run as the cathode sample for comparison (see Experimental section for more details). Figure S1 shows full XPS surveys of the TMF cathode, Fe reference, and LiF reference. In Figure S2(d) of the Fe 2p spectrum, the binding energies of Fe^0 are 704.7 and 717.7 eV of Fe-LiF cathode and 706.6 and 719.6 eV of the Fe reference sample for $2p_{3/2}$ and $2p_{1/2}$ respectively, which is in good agreement with the spin-orbit components split of $\Delta = 13.0$ eV.[1] In Figure S2(f), the F 1s spectra at 683.5 eV for the Fe-LiF cathode sample and 683.8 eV for the LiF reference sample can be assigned to F-M bonding, such as the F-Fe bond in FeF_x and the

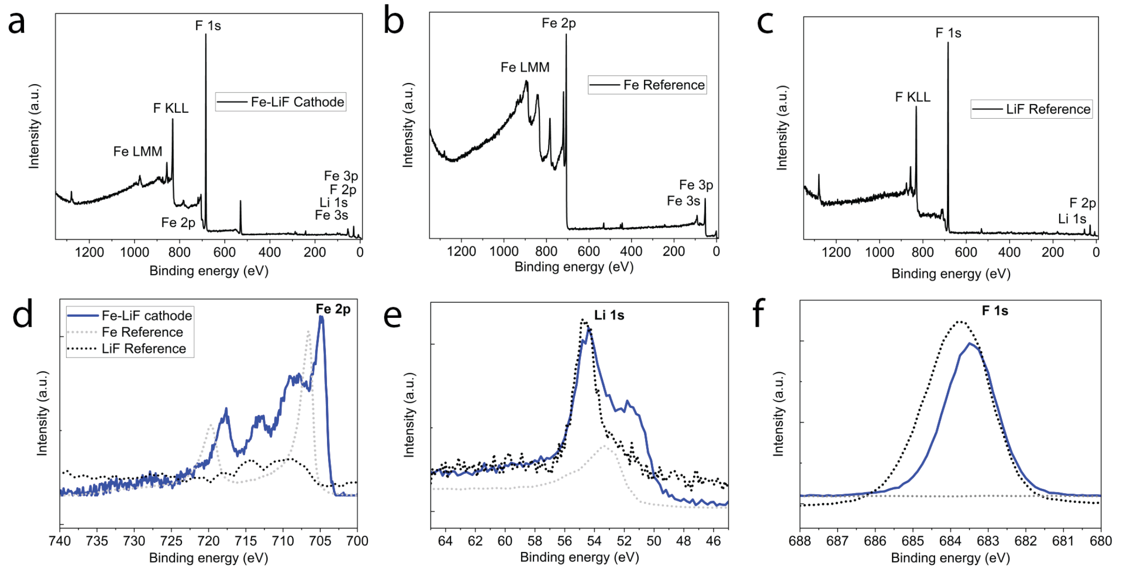

Figure S2: **(a)-(c)** XPS surveys of the TMF cathode, Fe reference and LiF reference, respectively.**(d)-(f)** XPS spectra of Fe-LiF cathode matching the characteristic lines for Fe, Li, and F. Reference measurements were conducted with in-house references (see Experimental section for more details).

F-Li bond in LiF.[2] These results indicate the presence of the LiF and FeF_x phases, which is consistent with the binding energy of 713.3 eV for the Fe-F bond in Fe 2p spectra and that of 54.5 eV for the Li-F bond in Li spectra (Figure S2(e)).[3,4] The XPS binding energy shows an approximate linear correlation with the local electrostatics around the excited atom, as noted for fluorides specifically.[5,6] Compared to iron, lithium has a lower positive charge due to its lower oxidation state. Therefore, the local electrostatic effects might be slightly weaker in pure LiF compared to LiF/FeF_x present in the Fe-LiF cathode. The lithium cation has a less pronounced influence on the electron density distribution around it than the iron cation in FeF_x. This may explain the shift of F 1s spectra towards the lower binding energies range and Fe 2p shift towards the higher binding energies for the Fe-LiF cathode vs. pure LiF reference.

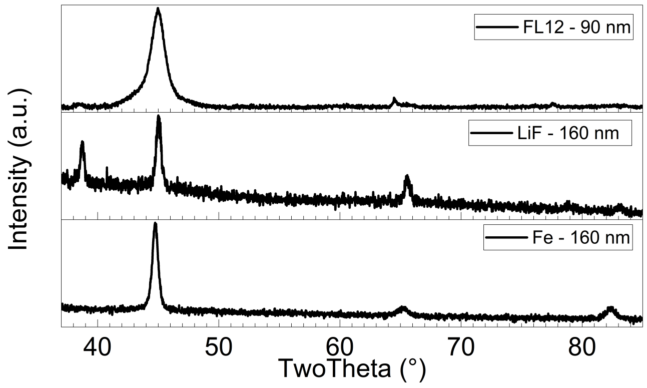

Figure S3: XRD diffractograms of the TMF cathode, LiF and Fe references. All layers were deposited using the same evaporator onto bare glass substrates. X-ray crystallography remains a difficult tool to use for such materials due to their heterogeneous nature and nano-sized domains. Furthermore, the crystallographic peaks of Fe and LiF mostly coincide, making peak analysis difficult.

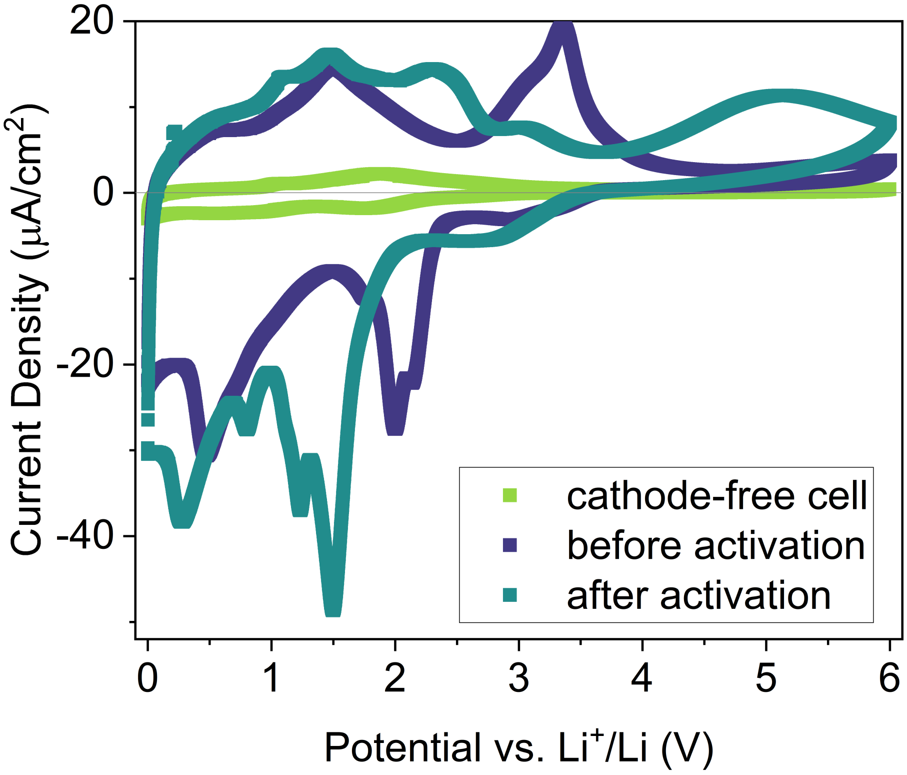

Figure S4: Cyclic voltammetry of the TMF cathode before activation and after activation as well as a reference cell constructed similarly to all cells while omitting the cathode layer. Cathode thickness is 70 nm. Scan rate 1 mV/s, 0 to 6 V.

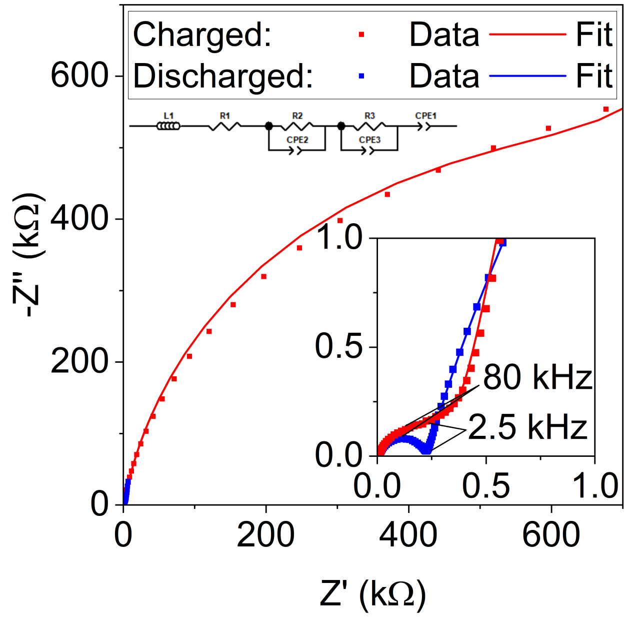

Figure S5: Electrochemical Impedance spectroscopy (EIS) of the TMF cathode in its charged and discharged state. EIS spectra were measured from 2 MHz to 0.5 Hz after charging and discharging the cathode at C/8 versus theoretical capacity. Inset is the high frequency region of the same data and also the equivalent circuit used to fit the data.

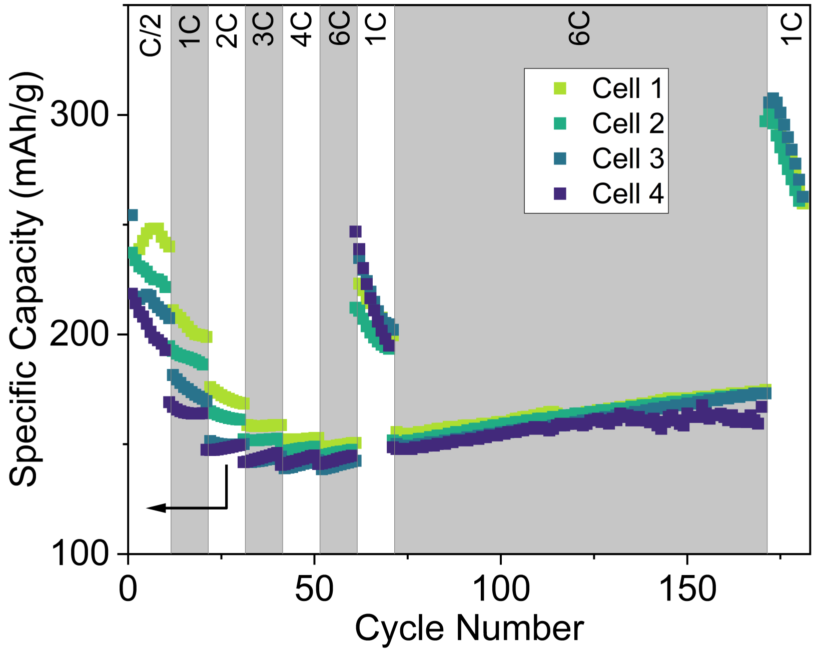

Figure S6: Statistical validation of specific discharge capacity of the Fe-LiF conversion cathode (90 nm thickness) at current densities ranging from 10 to 100 μA/cm^2 (C/2 to 6C).

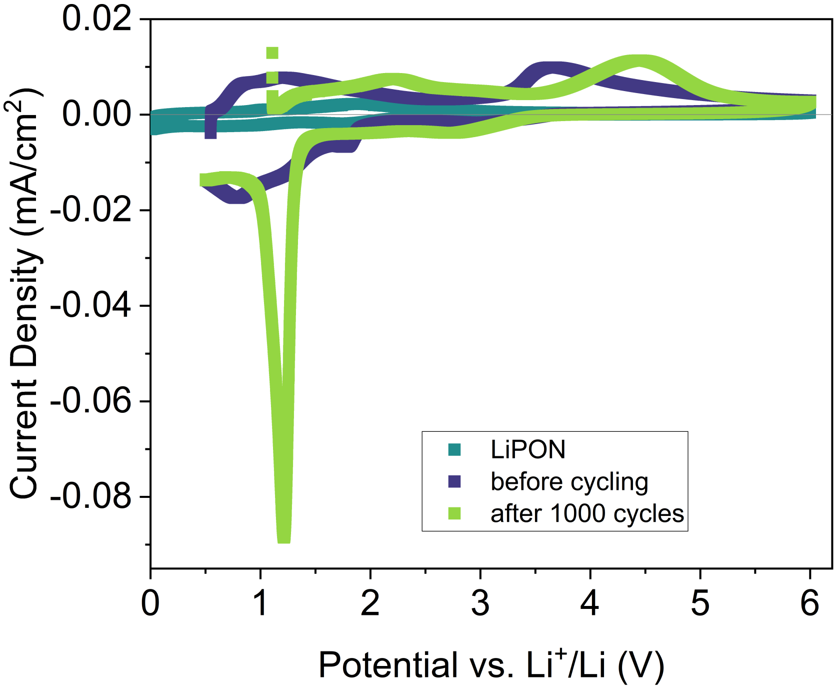

Figure S7: Cyclic Voltammetry of the TMF cathode before cycling and after 1000 cycles at 6C. The LiPON cyclic voltammetry is added as a reference. The TMF cathode cyclic voltammetry was conducted at 0.5 mV/s from 0.5 to 6 V and then back to 0.5 V. The LiPON measurements was conducted at 1 mV/s using the exact same cell structure (only omitting the cathode layer) from 0 to 6 V for 5 cycles. Only the 5th cycle is shown. The TMF cathode is 90 nm thick.

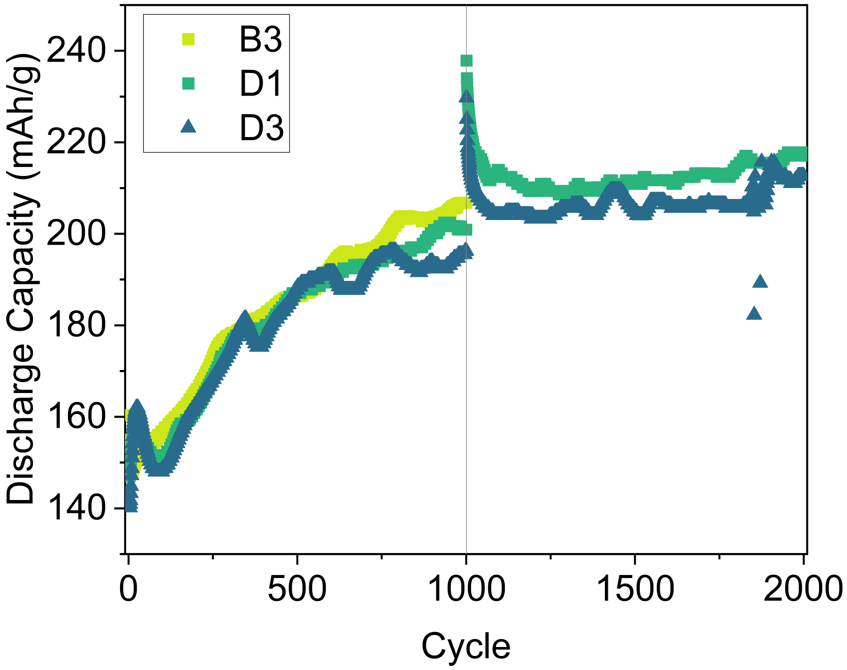

Figure S8: Discharge capacity versus cycle number of multiple cells with Fe-LiF cathodes cycling at 100 μA/cm^2 (6C vs. theoretical capacity of FeF_2).

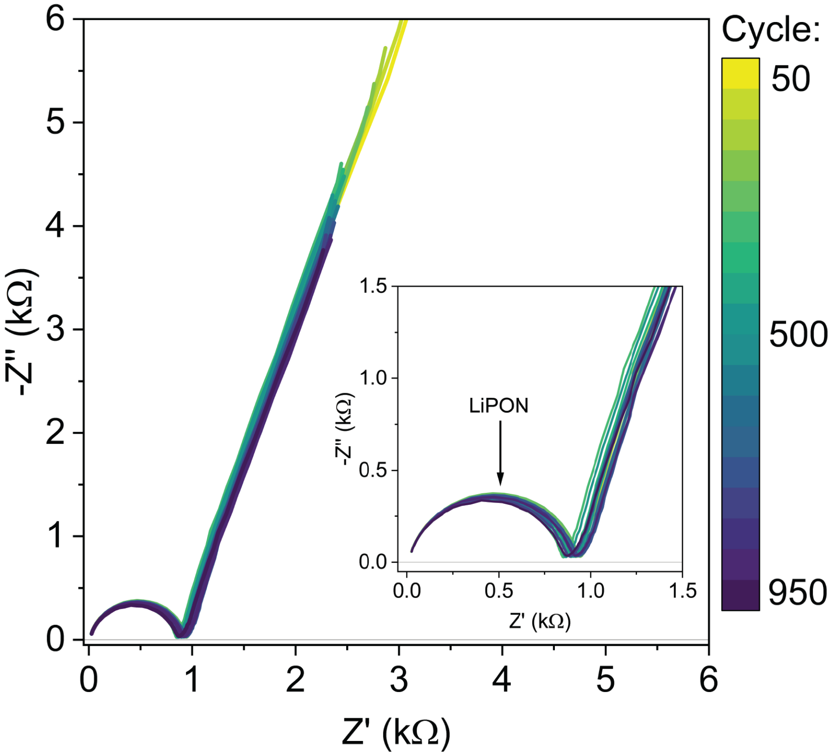

Figure S9: EIS spectra of a cell during long term cycling at 100 μA/cm^2 (6C vs. theoretical capacity of FeF_2). Over 1000 cycles, there is no evidence of LiPON degradation. The magnitude of the impedance at low frequency is decreasing as the cell is cycling, further validating the observed cathode activation.

References

(1) Biesinger, M. C.; Payne, B. P.; Grosvenor, A. P.; Lau, L. W.; Gerson, A. R.; Smart, R. S. Resolving surface chemical states in XPS analysis of first row transition metals, oxides and hydroxides: Cr, Mn, Fe, Co and Ni. *Applied Surface Science* **2011**, *257*, 2717–2730.

(2) Li, C.; Yin, C.; Mu, X.; Maier, J. Top-Down Synthesis of Open Framework Fluoride for Lithium and Sodium Batteries. *Chemistry of Materials* **2013**, *25*, 962–969.

(3) Zhao, T.; Li, L.; Chen, R.; Wu, H.; Zhang, X.; Chen, S.; Xie, M.; Wu, F.; Lu, J.; Amine, K. Design of surface protective layer of LiF/FeF3 nanoparticles in Li-rich cathode for high-capacity Li-ion batteries. *Nano Energy* **2015**, *15*, 164–176.

(4) Myung, S.-T.; Sakurada, S.; Yashiro, H.; Sun, Y.-K. Iron trifluoride synthesized via evaporation method and its application to rechargeable lithium batteries. *Journal of Power Sources* **2013**, *223*, 1–8.

(5) Kawamoto, Y.; Ogura, K.; Shojiya, M.; Takahashi, M.; Kadono, K. F1s XPS of fluoride glasses and related fluoride crystals. *Journal of Fluorine Chemistry* **1999**, *96*, 135–139.

(6) Sanz-Matias, A.; Roychoudhury, S.; Feng, X.; Yang, F.; Kao, L. C.; Zavadil, K. R.; Guo, J.; Prendergast, D. Excitonic Effects in X-ray Absorption Spectra of Fluoride Salts and Their Surfaces. *Chemistry of Materials* **2022**, *34*, 9144–9158.
